# The intestinal microbiota as an ally in the treatment of Alzheimer’s disease

**DOI:** 10.1017/gmb.2023.8

**Published:** 2023-06-19

**Authors:** Sabrina Sehn Hilgert, Daniel Penteado Martins Dias

**Affiliations:** Centro Universitário Barão de Mauá, Ribeirão Preto, Brazil

**Keywords:** Alzheimer’s, gut–brain axis, microbiota, pathophysiology, patients

## Abstract

The evolution of the understanding of the intestinal microbiota and its influence on our organism leverages it as a potential protagonist in therapies aimed at diseases that affect not only the intestine but also neural pathways and the central nervous system itself. This study, developed from a thorough systematic review, sought to demonstrate the influence of the intervention on the intestinal microbiota in subjects with Alzheimer’s disease. Clinical trials using different classes of probiotics have depicted noteworthy remission of symptoms, whose measurement was performed based on screenings and scores applied before, during, and after the period of probiotics use, allowing the observation of changes in functionality and symptomatology of patients. On the other hand, faecal microbiota transplantation requires further validation through clinical trials, even though it has already been reported in case studies as promising from the symptomatology point of view. The current compilation of studies made it possible to demonstrate the potential influence of the intestinal microbiota on Alzheimer’s pathology. However, new clinical studies with a larger number of participants are needed to obtain further clarification on pathophysiological correlations.

## Introduction

Research involving the gastrointestinal tract, and its systemic influences, have been developed for a long time. Still, in the 17th century, Antoni van Leeuwenhoek wrote about “animalcules” – bacteria – that he identified in his oral cavity with the help of his homemade microscopes, identifying differences between them and some bacteria that he observed in the faeces (Leeuwenhoek, 1683). The 19th century was marked by publications that boosted the foundations of perceptions of the interaction between microorganisms and the host, mainly addressing the role of the germ in disease, and the importance of microorganisms in physiology. The publication “A flora and fauna within living animals,” detailing anatomy, reproductive cycle, and specific aspects of “entozoa,” “ectozoa,” and “entophyte” of humans, was essential to induce research aimed at what would come to be defined as the microbiota (Leidy, 2012). Pasteur and collaborators, in 1879, described vibrios and their negative repercussions when present in wounds, supporting the “germ theory,” but also suggested that some microorganisms could be significant in human physiological processes, not only in pathologies (Pariente, 2019).

However, with the publication of the Henle–Koch postulates and the successful use of them to identify the relationship between a bacillus and tuberculosis (Koch, 1882), researchers at the time began to focus on the search for causative agents of pathology, which led to the “first golden age of microbiology.” Studying the beneficial relationship between microorganisms and humans remained in the background during this period, progressing more slowly until 1916. In that year, there was a correlation between the presence of specific bacterial strains and the inhibition of Salmonella replication, already known to be related to dysentery (Nissle, 1916). When he successfully isolated *Escherichia coli* from a soldier who had not succumbed to dysentery, Nissle began to cultivate it on agar and filled gelatine capsules with the cultures patenting the creation for the pharmaceutical industry at the time (Sonneborn, 2016). However, the first recognised use of the intestinal microbiota as a treatment method occurred in proving faecal transplantation effectiveness in treating patients with recurrent *Clostridium difficile* infection in 1958 (Eiseman et al*.,* 1958).

Research in this line of treatment has become increasingly frequent, especially with the increased antibiotic resistance in recent decades (Sonnenborn, 2016). The intensification of these researches, combined with new technologies, allowed the analysis of the influence of the intestinal microbiota beyond the barriers of the gastrointestinal tract, as well as the demonstration of the systemic influence on these microorganisms. Still in the 1970s, the ability of stress to alter the intestinal microbiota was demonstrated, considering it the causal factor of the observed differences (Tannock and Smith, 1972). In the clinical setting, the effect of stress on these microorganisms has already been demonstrated through the analysis of faecal content before and after the participation of volunteers in adverse situations with potential stressors (flight training and astronaut diet), evidencing the variation in the composition of the microbiota (Holdeman et al., 1976).

Although the first study to demonstrate that hormones produced in the central nervous system (CNS) are also found in the gastrointestinal tract was carried out in the 1930s (Euler and Gaddum, 1931), the term brain–gut axis took several decades to emerge. One of the first studies to use the term identified negative feedback on the major release of cholecystokinin (CCK) from the increase in the plasma concentration of this hormone, with the gastrointestinal tract as a possible modulator since the substance does not cross the blood–brain barrier (Banks, 1980). Moreover, in this way, the existence of the afferent pathway of the brain–gut axis was evidenced.

The proof of the existence of the efferent pathway occurred years later. In the year 2000, there was a flood that contaminated drinking water in a city in Canada, infecting part of the population with microorganisms, such as *Campylobacter jejuni.* This population was evaluated, and 8 years later, 2,451 individuals completed the reassessment, out of a total of 4,561 who became infected, and approximately half of the re-evaluated participants (1,166 individuals) were diagnosed with irritable bowel syndrome, having as independent risk factors anxiety and depression (Marshall et al., 2010). Another research, looking for treatment alternatives for hepatic encephalopathy, demonstrated that the oral administration of a low-spectrum antibiotic with the main action on enterobacteria could reverse hepatic encephalopathy more efficiently than the intravenous route. It was seen remission of behavioural symptoms, as well as normalisation of laboratory tests, improvement of mental status, and intellectual abnormalities in patients with decompensated liver disease (Schiano, 2010).

Seeking greater insight into the subject, an experimental study conducted by Neufeld et al. (2011) compared brain development and behavioural expression between germ-free (GF) mice (i.e., animals that do not have contact or colonisation by normal microbiota) and rats raised in an environment free of specific pathogens (SPF; i.e., animals colonised by the normal microbiota and isolated from contact with disease-causing microorganisms). The results showed divergences, demonstrating that the SPF population showed behaviour similar to anxiety, which did not occur in GF animals. In addition, there were differences in the central expression of genes, culminating in modifications in the amygdala, hippocampus, and dentate gyrus, areas involved with behaviour and composers of the intrinsic signalling pathway of the CNS responsible for triggering feelings such as fear, anxiety, and the fight or flight response. When subjected to the SPF-rearing environment, the GF population did not present anxiety-like behaviour, only their offspring, which were reared in the same environment as the SPF. These analyses were able to demonstrate that the differences in the CNS level in the SPF rats were related to the composition of their intestinal microbiota (Neufeld et al., 2011). Heijtz et al. (2011) obtained similar results, showing that the GF animals explored the environment they were exposed to more inadvertently and extensively when compared to the SPF group, which showed greater caution in the unknown environment. A new population of GF rats was obtained and inoculated with the same microbiota as the SPF group shortly after birth, forming the group of conventionalised adults (CON). Their behaviour was similar to the SPF population after undergoing the same tests. Research indicates that GF mice showed lower activation of genes related to fear and anxiety in cortical regions such as the hippocampus, cingulate cortex, and amygdala (Heijtz et al., 2011).

In the clinical field, a double-blind trial with 55 properly randomised participants used probiotics (BP) and placebo (PL) for 30 days, seeking to compare levels of anxiety and depression between the groups through questionnaires. After the treatment period (30 days), only the PB group showed an improvement in the psychological distress score, as well as in the self-blame score and higher score in problem-solving in addition to a decrease in the mean urinary cortisol level. The comparison was performed with results obtained in tests before the period of administration of PB or PL. The probiotics used included the genera *Lactobacillus* and *Bifidobacterium* (Messaoudi et al., 2011).

The identification and subsequent understanding of the neural axis responsible for this neurotransmission took place through the observation that vagotomised rats do not present behavioural or neurochemical differences after the use of probiotics, pointing out the vagus nerve as an important communication route between the intestinal microbiota and the CNS (Bravo et al., 2011). Although the vagus nerve is the main extrinsic neural pathway from the CNS to the intestine, other communication pathways have already been demonstrated. It is a bidirectional connection in which the CNS can interact with the intestine, and the intestine with the CNS (Wang and Wang, 2016), through the vagus nerve itself, spinal nerves, other divisions of the autonomic nervous system, endocrine and immune pathways (cytokines), in addition to other pathways that are still under investigation (Margolis et al*.,* 2021). Brain imaging has already demonstrated this reciprocal activation, in which intestinal stimuli simultaneously activate crucial brain regions involved in emotion regulation (Mayer, 2011).

In addition, under clinical aspects, symptoms of gastrointestinal disorders have been associated with psychological disorders and psychiatric diagnoses. More than that, significant diseases such as Parkinson’s can cause dysfunction in the gastrointestinal tract even before neurological symptoms are evident (Bove and Travagli, 2019). In this context, the intestinal microbiota also began to be investigated, especially after the publication of the aforementioned study on the use of oral antibiotics for the treatment of hepatic encephalopathy. Among the pathways of influence of the microbiota on the CNS, it has been shown that 90% of the body’s serotonin is produced by intestinal cells through signalling by compounds metabolised by bacteria, culminating in the activation of central nervous areas through the conduction of the stimulus by the vagus nerve afferent (Reigstad et al., 2015; Yano et al., 2015). The intestinal microbiota is also responsible for helping to maintain the intestinal mucus layer, so changes in the microbiota induced by inadequate diet cause compromise of the mucus layer, allowing access to pathogens to dendritic cells. Due to this exposure, immune mediators are released into the systemic circulation, which can cause immune activation in different locations, including the CNS (André et al*.,* 2019). This activation correlates with neurodegenerative disease pathophysiology (Margolis et al., 2021).

With this in mind, researchers have developed experimental designs involving transgenic animals with Alzheimer’s disease (AD), seeking to improve symptoms from changes in the intestinal microbiota through probiotics and faecal microbiota transplantation (FMT). The results indicate that probiotic supplementation in mice decreased β-amyloid plaques in the hippocampus, when combined to exercises (Abraham et al., 2019). In addition, probiotic treated mice show reduced inflammation and permeability of the intestinal wall, as well as a tendency to normalise inflammatory modulators in the systemic circulation, although central-level effects on the reduction of β-amyloid plaques, cytokine levels and gliosis were absent (Kaur et al., 2020). FMT also affects these animals since the transplantation of microbiota from healthy animals to models with Alzheimer’s caused a decrease in the number of central B-amyloid plaques, improvement in cognition, and reversal of abnormalities in the expression of genes that modulate the activity of macrophages (Kim et al., 2020; Dodiya, 2021). Furthermore, the Federal Drug Administration (FDA) recently approved the rectal administration of a probiotic suspension to prevent recurrent *C. difficile* infection. The technique is also more cost-effective when compared to traditional treatment (i.e., prolonged use of antibiotics, without subsequent administration of the probiotic suspension; Lodise et al., 2023; Walter and Shanahan, 2023).

Due to the beneficial effects of healthy microbiota on the brain–gut axis and its repercussion on AD symptoms, already demonstrated in extensive literature with animal models, intestinal microbiota emerges as a potential ally in treating AD patients.

## Objectives

The objective of the present study was to search the literature for correlations between alterations caused in the intestinal microbiota of patients with AD and the consequent change in the clinical profile of patients through the superposition of data obtained in a systematic literature review.

## Specific Objectives

Identify in the literature and overlap the results of recent clinical studies that test the efficacy of the use of probiotics and FMT in patients with Alzheimer’s.

Describe the data found in order to objectively expose the results obtained from each study using probiotics or FMT.

Correlate the results found with pathophysiological hypotheses that seek to explain the results obtained in the studies described to prove or rule out the effectiveness of the use of probiotics or FMT in patients with AD.

## Methods

A systematic literature review was conducted using the PubMed (https://pubmed.ncbi.nlm.nih.gov) and CAPES journals (https://www-periodicos-capes-gov-br.ezl.periodicos.capes.gov.br) databases. The search keywords were: microbiota, Alzheimer’s, gut–brain, microbiota–gut–brain, probiotics, FMT, and microbiome. The results were restricted between 2015 and 2022 since clinical trials involving patients with Alzheimer’s and intervention on intestinal microbiota were not carried out before that period, only using an animal model. The filters “articles” and “peer-reviewed journals” were also used. In all, 7,050 articles were found. Of these, 471 articles were selected after reading the titles and abstracts, as they fit the theme focused on intervention in the intestinal microbiota in patients with AD. These articles were then submitted to the following inclusion and exclusion criteria based on their methodology: the articles should be empirical studies, excluding reviews and study protocols; studies should be clinical trials or case reports, excluding studies performed in animal models; duplicates were excluded. Through these criteria, 18 articles were selected. A careful reading of the 18 articles was carried out is considered suitable for this systematic review those in which there was an adequate description of the intervention in the intestinal microbiota through the use of probiotics or transplantation of faecal microbiota in patients with AD, having as an evaluation method of the variation of the patient’s symptomatology objective questionnaires approved for this purpose. Finally, five articles met these requirements and were described in this systematic review.

## Results and discussion

In the clinical trial developed by Leblhuber et al. (2018), 20 AD patients were subjected to the mini-mental state examination (MMSE) and the Clock Drawing Test (CDT), before and after intervention with probiotics. For 28 days, patients regularly used probiotics containing *Lactobacillus* sp., *Lactococcus lactis,* and *Bifidobacterium* sp. After treatment ceased, no changes were observed in the MMSE and CDT scores. It was concluded that, despite increased serum levels of inflammatory markers, no substantial changes were seen on the patients’ cognitive levels, possibly due to the short duration and small sample investigated.

Subsequently, Hazan (2020) clarified the case of an 82-year-old man with AD and recurrent *C. difficile* infection. The patient was evaluated through the MMSE before and after the FMT procedure using the Borody method. After 8 weeks, there was an increase in the MMSE score of +6 points. In addition, the wife also reported improvement in mental acuity and mood. After 16 weeks, there was an improvement in memory capacity and no negative progression of Alzheimer’s symptoms. Finally, after 24 weeks, the MMSE score increased by +9 from baseline; improvements in mood, social interaction, and affection were also observed. In the final considerations, the author explained that due to the potential role of the intestinal microbiome in the pathogenesis of Alzheimer’s, microbiome modulation represents a promising treatment route.

More recently, Park et al. (2021) followed a 90-year-old woman with AD, systemic arterial hypertension (SAH), type II diabetes mellitus (DMII), chronic kidney disease (CKD), and recurrent *C. difficile* infection, who required perform FMT for the treatment of reinfections. Before the procedure, she was assessed using the MMSE, Montreal Cognitive Assessment (MoCA), and Clinical Assessment of Dementia (ACD). After 4 weeks of FMT, she showed an increase of +3 in MMSE (baseline 15), +1 in MoCA (baseline 11), and +0 in ACD (baseline 1). After 12 weeks, the scores changed again from baseline: MMSE +5, MoCA +5, and ACD -0.5. It is concluded that the case offers evidence of the benefits of FMT in Alzheimer’s patients. It also suggests an association between the gut microbiome and cognitive function.

In a recent clinical trial conducted by Akhgarjand et al. (2022), 90 patients with AD were sorted out into three groups using age and sex criteria. Firstly, all individuals were subjected to the MMSE for illiterate patients, to the categorical test of verbal fluency (CFT) and to the evaluation of the performance of daily activities through the Barthel Index (BI). Over the next 12 weeks, one group received *Bifidobacterium longum*, another received *Lactobacillus rhamnosus* HA-114, and the last group received placebo. Following the intervention period, MMSE was found higher in both *Bifidobacterium* and *Lactobacillus* groups, when compared to baseline. No changes were observed in MMSE of placebo group, as compared to baseline. CFT was found higher in both intervention groups, *Bifidobacterium* and *Lactobacillus*, when compared to baseline and compared to placebo. Finally, BI was found similar following 12 weeks of treatment in both *B. longum* and *L. rhamnosus* groups. All parameters assessed remained unchanged in placebo groups following the 12-week period. Authors concluded that the use of probiotics, as an adjuvant, has benefits on disease progression and quality of life of patients with AD.

Although most studies assessing the effects of probiotics therapy in AD patients have short-duration protocols and involve small groups of individuals, some hypotheses may be raised. Seeking to correlate the data presented, we must remember that AD is a neurodegenerative pathology that has shown an increase in prevalence in recent years (Doifode et al., 2021). It mainly affects the elderly over 65, ranking first among the causes of dementia (Alzheimer’s Association, 2019). It begins its symptoms insidiously, first affecting learning and memory, progressing to deficits in attention, language, and social behaviour. After the first symptoms, the carrier usually clinically involutes for approximately 5 to 12 years, when, inevitably, he dies (Long and Holtzman, 2019). In addition to the direct impact on the patient’s quality of life, family members and caregivers are emotionally and financially affected as the patient develops irreversible dependence (Doifode et al., 2021). The main responsible for all these described conditions is the β-amyloid peptide (PβA), which, when deposited in the extracellular epithelial tissue of the brain, aggregates and gives rise to amyloid neuritic plaques. It then affects synaptic activity and local capillary blood flow as it binds to oligomers and fibrils, preventing them from acting normally in these functions (Long and Holtzman, 2019). However, ironically, there have been recent reports that PβA is produced on demand from signals from the immune system, acting as a defence, precisely because it performs extracellular aggregation (Wang et al., 2021). In addition to PβA, the increase in tau protein phosphorylation, involved in the regulation of axonal transport and stabilisation of neuronal microtubules, also contributes to the progression of the disease. Hyperphosphorylation leads to changes that culminate in the formation of neurofibrillary tangles, making synapses even more complex and making axonal transport insufficient (John and Reddy, 2021). From the deposition of PβA, there is an induction of oxidative stress, which increases the phosphorylation of the tau protein (Belkouch et al., 2016).

Regarding PβA aggregation, it was shown that the greater the production of different types of β-amyloid protein, the more intensely and earlier the aggregation occurs. Physiologically, the human organism can produce around 30 different types of amyloidogenic proteins. However, depending on its composition, the intestinal microbiome can contribute to the production of more subtypes, increasing aggregation (Sampson et al., 2020). In addition, the use of antibiotic cocktails in transgenic mice and rats carrying “APP SWE” and “PS1 L166P” – family genes linked to Alzheimer’s – caused less progression of amyloid plaques in male brains when compared to the group that did not receive the cocktail, in addition to reduced neuroinflammatory activity, mediated by microglia. The change in the microbiome also led to an increase in anti-inflammatory substances in the plasma and a decrease in pro-inflammatory cytokines (Minter et al., 2016; Dodiya et al., 2019). There was also a difference in the gene expression induced by the microbiota between the group treated with antibiotics and the untreated group. When performing FMT from the untreated group to the treated group, partial restoration of the pathology by PβA deposition was described (Dodiya et al., 2019). Probiotics also can alter the inflammatory response, as observed by Leblhuber et al. (2018). The researchers observed an increase in serum inflammatory markers, probably due to the activation of macrophages, questioning whether this modulation could delay or accelerate neurodeterioration in patients with AD, depending on the level of immune response triggered (Leblhuber et al., 2018). Harach et al. (2017) also evidenced differences in central PβA deposition when comparing GF and conventional mice, both transgenic for the mutations linked to Alzheimer’s. In addition to reduced plaque in the GF mice, decreased cortical inflammation and increased enzymes that degrade PβA were also demonstrated. However, in the long term, the use of antibiotics causes changes in the morphology and reactivity of microglia, primarily responsible for physiological neuroinflammatory responses in the CNS and a decrease in astrocyte reactivity, which also contribute to the neuroinflammatory response (Minter et al., 2016). It has already been shown that the intestinal microbiota is responsible for the constant physiological modulation and maturation of microglia in the CNS (Erny et al., 2015).

## Conclusion

Changes in the intestinal microbiota from probiotics and FMT are shown to be effective in improving the symptoms of AD when used as an adjunct to the treatment of already established drugs, usually when medications do not achieve sufficient symptom control. However, FMT still requires clinical trials with larger samples to demonstrate the safety of the procedure in patients with AD since the few case reports in patients with recurrent infection by associated *C. difficile* are insufficient to establish efficacy and safety to apply the therapy in AD patients widely.

## Data Availability

Data used and presented for the preparation of the systematic analysis are available on the drive at https://drive.google.com/drive/folders/1NvCLbyfqpHtQwjUxhpOUxlhtZm5-inZn?usp=sharing.
